# Spatiotemporal and quantitative analyses of phosphoinositides – fluorescent probe—and mass spectrometry‐based approaches

**DOI:** 10.1002/1873-3468.70200

**Published:** 2025-10-22

**Authors:** Hiroaki Kajiho, Shin Morioka, Junko Sasaki, Takehiko Sasaki

**Affiliations:** ^1^ Department of Biochemical Pathophysiology, Medical Research Laboratory, Institute of Integrated Research Institute of Science Tokyo Bunkyo‐ku Tokyo Japan; ^2^ Department of Lipid Biology, Graduate School of Medical and Dental Sciences Institute of Science Tokyo Bunkyo‐ku Tokyo Japan

**Keywords:** fluorescent probe, intracellular localization, lipidomics, mass spectrometry, PH domain, phosphoinositide

## Abstract

Comprehensive understanding of phosphoinositide signaling requires both spatiotemporal visualization and precise quantitative analysis of individual lipid species. Phosphoinositides, a family of phosphorylated derivatives of phosphatidylinositol (PI), are structurally diverse lipid messengers that orchestrate a wide range of cellular functions, including membrane trafficking, cytoskeletal dynamics, and signal transduction. Due to their dynamic metabolism and compartment‐specific localization, their analysis demands complementary strategies that integrate live‐cell imaging with molecular quantification. In this review, we first summarize the development and application of fluorescence‐based probes designed to monitor the distribution and dynamics of phosphoinositides in living cells, highlighting their specificity, targeting mechanisms, and limitations. We then provide an overview of recent advances in mass spectrometry‐based methodologies that enable high‐sensitivity, isomer‐resolved quantification of phosphoinositides in biological specimens, including improvements in lipid extraction, derivatization, and chromatographic separation. Together, these dual approaches offer synergistic insights into the biochemical and cellular regulation of phosphoinositide signaling.

## Graphical abstract

Fluorescent probes allow dynamic visualization of phosphoinositides in living cells (left), whereas mass spectrometry provides high‐sensitivity, isomer‐resolved quantitation (right). Their synergistic use captures complementary aspects of lipid signaling. This review illustrates how these approaches reveal the spatiotemporal regulation and quantitative dynamics of phosphoinositides. Portions of this figure were adapted and modified from Morioka *et al.*, Nat. Commun., 2022, CC BY 4.0.

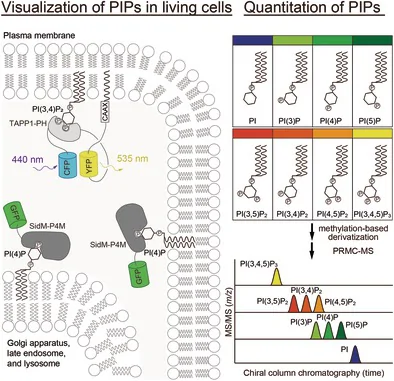

## Abbreviations

**ARF**, ADP‐ribosylation factor

**Atg**, autophagy‐related protein

***Bc*PI‐PLC**, *Bacillus cereus*‐derived PI‐specific phospholipase C

**Btk**, Bruton's tyrosine kinase

**CAD**, collisionally activated dissociation

**CERT**, ceramide transfer protein

**Ci‐VSP**, *Ciona intestinalis* voltage sensor‐containing phosphatase

**DAN**, 2‐dimethylamino‐6‐acyl‐naphthalene

**ddFP**, dimerization‐dependent fluorescent protein

**DEAE**, diethylaminoethyl

**DFCP**, double FYVE‐containing protein 1

**DIA**, data‐independent acquisition

**EEA1**, early endosomal antigen 1

**EGF**, epidermal growth factor

**ENTH**, epsin N‐terminal homology

**ER**, endoplasmic reticulum

**ESI**, electrospray ionization

**FAPP1**, four‐phosphate‐adaptor protein 1

**FRET**, Förster resonance energy transfer

**FYVE**, Fab1, YOTB1, Vac1, and early endosomal antigen 1

**Grp1**, general receptor for 3‐phosphoinositides 1

**GEF**, guanine nucleotide exchange factor

**HPLC**, High Performance Liquid Chromatography

**Hrs**, hepatocyte growth factor‐regulated tyrosine kinase substrate

**IMS**, imaging mass spectrometry

**ING2**, inhibitor of growth protein 2

**LC**, liquid chromatography

**MAG**, monoacylglycerol

**ML1N**, the cytosolic N‐terminal polybasic region of mucolipin‐1

**MRM**, multiple reaction monitoring

**MS**, mass spectrometry

**MS/MS**, tandem mass spectrometry

**NO**_**2**_ **BF**_**4**_, nitronium tetrafluoroborate

**OSBP**, oxysterol‐binding protein

**OSH**, oxysterol‐binding protein homologs

**PC**, phosphatidylcholine

**PE**, phosphatidylethanolamine

**PH**, pleckstrin homology

**PHD**, plant homeodomain

**PI**, phosphatidylinositol

**PI(3)P**, phosphatidylinositol 3‐phosphate

**PI(4)P**, phosphatidylinositol 4‐phosphate

**PI(5)P**, phosphatidylinositol 5‐phosphate

**PI(3,4)P**_**2**_, phosphatidylinositol 3,4‐bisphosphate

**PI(3,5)P**_**2**_, phosphatidylinositol 3,5‐bisphosphate

**PI(4,5)P**_**2**_, phosphatidylinositol 4,5‐bisphosphate

**PI(3,4,5)P**_**3**_, phosphatidylinositol 3,4,5‐trisphosphate

**PIKfyve**, phosphoinositide kinase, FYVE‐type zinc finger containing

**PLCδ1**, phospholipase C delta 1

**PRMC‐MS**, phosphoinositide regioisomer measurement by chiral column chromatography and mass spectrometry

**PS**, phosphatidylserine

**PX**, phagocyte oxidase homology

**P4M**, PI(4)P‐binding module

**p40phox**, PX domain from neutrophil cytosol factor 4, 40 kDa

**SFC**, supercritical fluid chromatography

**SH2**, Src homology 2

**SHIP**, SH2‐domain‐containing inositol polyphosphate 5‐phosphatase

**SnxA**, sorting nexin A

**SRM**, selected reaction monitoring

**SWATH‐MS**, sequential windowed acquisition of all theoretical fragment ion mass spectra

**TAPP1**, tandem PH‐domain‐containing protein 1

**Tiam1**, T‐lymphoma invasion and metastasis 1

**TMS**, trimethylsilyl

**TRPML1**, the transient receptor potential cation channel, mucolipin subfamily

**Vac**, vacuolar segregation protein

**YOTB1**, SPbeta prophage‐derived uncharacterized protein

**WCB**, (E)‐N′‐((7‐(diethylamino)‐9,9‐dimethyl‐9H‐fluoren‐2‐yl)methylene)acrylohydrazide

**WCR**, 3‐((acryloyloxy)imino)‐2‐((7‐(diethylamino)‐9,9‐dimethyl‐9H‐fluoren‐2yl)methylene)‐2,3‐dihydro‐1H‐inden‐1‐one

Phosphoinositides, are a family of phosphorylated derivatives of phosphatidylinositol (PI), a low‐abundance yet functionally critical class of membrane lipids localized predominantly to the cytoplasmic leaflet of cellular membranes [1]. Despite their modest abundance compared with structural lipids, phosphoinositides play indispensable roles in a wide range of cellular processes, including membrane trafficking, cytoskeletal dynamics, cell polarity, and signal transduction [2, 3, 4]. Minor fluctuations in the localization or abundance of specific phosphoinositide species can elicit profound biological responses and are increasingly implicated in the pathogenesis of cancer, neurodegenerative disorders, and metabolic syndromes [5, 6, 7].

**Fig. 1:**
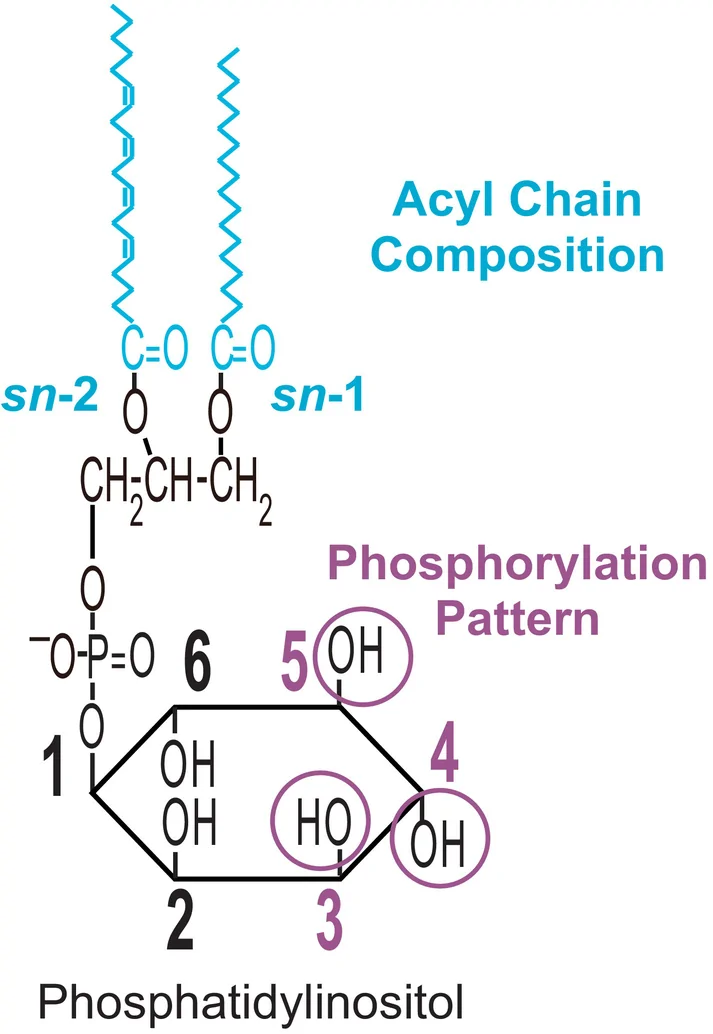
Structural diversity of phosphoinositide. In mammalian cells, hundreds of phosphoinositide molecular species are generated through eight phosphorylation patterns of the inositol headgroup (magenta), combined with variations in the length and degree of unsaturation of the fatty acyl chains at the stereospecific numbering *sn‐*1 and *sn‐*2 positions (cyan).

Phosphoinositides are defined by the phosphorylation status of the 3‐, 4‐, and 5‐hydroxyl positions of the inositol ring, giving rise to seven major species: phosphatidylinositol 3‐phosphate [PI(3)P], phosphatidylinositol 4‐phosphate [PI(4)P], phosphatidylinositol 5‐phosphate [PI(5)P], phosphatidylinositol 3,4‐bisphosphate [PI(3,4)P_2_], phosphatidylinositol 3,5‐bisphosphate [PI(3,5)P_2_], phosphatidylinositol 4,5‐bisphosphate [PI(4,5)P_2_], and phosphatidylinositol 3,4,5‐trisphosphate [PI(3,4,5)P_3_; PIP_3_], in addition to the unphosphorylated PI backbone (Fig. 1). These species are nonuniformly distributed across cellular membranes and undergo rapid, reversible interconversion by tightly regulated kinases and phosphatases, making their study particularly challenging.

A central goal in phosphoinositide research has been to visualize their spatiotemporal dynamics in living cells and to quantify their molecular diversity with high precision. Genetically encoded biosensors, often composed of lipid‐binding modules, such as pleckstrin homology (PH), phosphatidylinositol 3‐phosphate 5‐kinase (Fab1), SPbeta prophage‐derived uncharacterized protein (YOTB1), vacuolar segregation protein PEP7 (Vac1), and early endosomal antigen 1 (EEA1) (FYVE) or epsin N‐terminal homology (ENTH) domains fused to fluorescent proteins, have enabled real‐time imaging of specific phosphoinositide species in live cells [8, 9, 10, 11, 12]. These probes have revolutionized our understanding of the subcellular localization and stimulus‐induced dynamics of individual species. However, probe design requires a careful balance between affinity and specificity, and many probes remain blind to structural heterogeneity at the level of fatty acyl chains, potentially overlooking molecular diversity among phosphoinositide variants.

In parallel, advances in mass spectrometry (MS) have enabled extremely sensitive, isomer‐resolved quantification of phosphoinositide molecular species, including variants differing in fatty acyl composition (Fig. 1). Modern techniques combining chemical derivatization, optimized extraction protocols, and sophisticated liquid chromatography (LC)–tandem MS (MS/MS) platforms now allow for absolute quantification of more than 200 distinct acyl variants across all eight phosphoinositide classes. These MS‐based approaches complement imaging‐based strategies by providing molecular‐level resolution and broad applicability across diverse biological samples, from subcellular compartments to intact tissues.

In this review, we first summarize the design, advantages, and limitations of intracellular fluorescent probes for phosphoinositide detection. In the latter sections, we detail recent developments in MS‐based techniques for quantitative phosphoinositide analysis, highlighting how these complementary approaches together provide a powerful framework for understanding the spatiotemporal and molecular complexity of phosphoinositide biology.

## Visualization of phosphoinositide dynamics using fluorescent probes

To provide a structured overview of the phosphoinositide probes discussed in this review, Table 1 summarizes their molecular origin, target specificity, and characteristic subcellular distribution.

**Table 1:** Intracellular localization probes for phosphoinositides.

Probe name	Target	Origin	Localization	References
Clickable myo‐inositol	PI	Synthetic analogs	Broad	[13]
*Bc*PI‐PLC	PI	PI‐PLC (*Bacillus cereus*)	Golgi, ER, mitochondria, and peroxisome	[14]
2xFYVE	PI(3)P	Hrs	Early endosome	[15]
EEA1‐FYVE	PI(3)P	EEA1	Early endosome	[8]
DFCP1	PI(3)P	PI(3)P‐binding region of DFCP1	Omegasome	[16, 17]
SidM‐P4M	PI(4)P	SidM	Golgi, plasma membrane, late endosome/lysosome	[18]
P4Mx2	PI(4)P	SidM	Golgi, plasma membrane	[19]
3xTAPP1‐PH	PI(3,4)P_2_	TAPP1	Early endosome and lysosome	[20]
PLCδ1‐PH	PI(4,5)P_2_	PLCδ1	Plasma membrane	[21, 22, 23]
ENTH	PI(4,5)P_2_	Epsin	Plasma membrane and cytoplasm	[24]
SnxA PX	PI(3,5)P_2_	SnxA (*Dictyostelium discoideum*)	Phagosome and macropinosome	[25, 26]
Grp1‐PH	PIP_3_	Grp1	Plasma membrane	[27]
Btk‐PH	PIP_3_	Btk	Plasma membrane	[28]

### Fluorescent probes for phosphatidylinositol (PI)

Phosphatidylinositol (PI) serves as the precursor for all other phosphoinositide species and plays a pivotal role in their biosynthesis [1]. Its relatively uniform distribution across various intracellular membranes has posed a significant challenge for direct visualization.

Traditional visualization techniques for PI have relied primarily on indirect methods, such as metabolic labeling followed by lipid extraction and MS. However, recent advances have introduced metabolically incorporated myo‐inositol analogs coupled with click chemistry [13], allowing for fluorescent tagging and visualization of total PI pools in living cells. Although this approach does not distinguish PI from its phosphorylated derivatives, it will provide a snapshot of global phosphoinositide metabolism.

Additionally, *Bacillus cereus*‐derived PI‐specific phospholipase C (*Bc*PI‐PLC) is a potential PI probe. *Bc*PI‐PLC was originally identified for its ability to hydrolyze PI on unilamellar vesicles [29]. Expression of EGFP–*Bc*PI‐PLC in cells uncovered that PI is not uniformly distributed, but instead enriched at specific intracellular membranes, such as the Golgi and the endoplasmic reticulum (ER) [14]. These findings suggest that the ER may serve as the primary site for the initiation of phosphoinositide biosynthesis.

Despite these advances, specific and live‐cell‐compatible probes for unmodified PI remain scarce. Future directions may involve engineering novel protein domains with improved specificity toward the PI headgroup.

### Fluorescent probes for phosphatidylinositol 3‐phosphate [PI(3)P]

PI(3)P is enriched on early endosomes and plays a central role in endosomal trafficking, membrane tethering, and autophagy [30, 31]. The FYVE domain has become the most widely used probe for PI(3)P [8]. A common construct is 2xFYVE–GFP from hepatocyte growth factor‐regulated tyrosine kinase substrate (Hrs), which uses two tandem FYVE domains to improve binding avidity and localization fidelity [15]. This construct localizes robustly to early endosomes and is widely validated in various cell types. However, overexpression of FYVE domains may perturb endosomal function, and care must be taken to control expression levels.

Alternative probes include the phagocyte oxidase homology (PX) domain from neutrophil cytosol factor 4, 40 kDa (p40phox), which also binds PI(3)P but with different membrane affinity and dynamics than the FYVE domain [32, 33]. This has enabled complementary labeling in situations where FYVE domain behavior may be limiting. Another important probe is double FYVE‐containing protein 1 (DFCP1; also known as zinc finger FYVE domain‐containing protein 1), a protein that contains tandem FYVE domains and localizes to omegasomes, ER‐derived PI(3)P‐enriched membrane subdomains implicated in autophagy initiation [16]. GFP–DFCP1 constructs have been used to visualize PI(3)P dynamics specifically during autophagosome formation [17]. Unlike 2xFYVE, DFCP1 exhibits a distinct localization pattern, including lipid droplets [34], and may provide better resolution of ER‐associated PI(3)P pools. However, DFCP1's own role in autophagy or lipid droplet formation may complicate interpretations of its probe utility.

Overall, the FYVE domain remains the gold standard for PI(3)P detection, but additional tools—including PX domains and DFCP1—would offer complementary insight and reduce potential artifacts.

### Fluorescent probes for phosphatidylinositol 4‐phosphate [PI(4)P]

PI(4)P plays a vital role in Golgi function, vesicular trafficking, and the regulation of lipid and protein sorting. PI(4)P is found not only at the Golgi apparatus, but also at the plasma membrane and endosomes, although its relative abundance and functional roles vary by compartment.

Historically, the PH domains of four‐phosphate‐adaptor protein 1 (FAPP1; also known as pleckstrin homology domain‐containing family A member 3) and oxysterol‐binding protein (OSBP) were among the first tools used to visualize PI(4)P [35, 36, 37, 38]. Similarly, ceramide transfer protein (CERT) and oxysterol‐binding protein homolog (OSH) proteins in yeast, which also harbor PH domains, were employed as PI(4)P probes [38, 39, 40]. These proteins recognize PI(4)P‐enriched membranes—primarily Golgi apparatus—and have been instrumental in highlighting PI(4)P function in lipid exchange between organelles [41]. However, these probes often depend on protein–protein interactions, such as those with ADP‐ribosylation factor 1 (ARF1) or other small GTPases, for their correct localization [37, 38].

A significant advancement came with the identification of P4M, a PI(4)P‐binding module derived from the bacterial multifunctional virulence effector protein SidM, also referred to as DrrA, as well as its improved versions, such as P4Mx2, which contains tandem P4M domains [18]. P4M binds PI(4)P with high affinity and targets the Golgi apparatus, Rab7‐positive late endosomes/lysosomes, and plasma membrane, offering broader coverage of cellular PI(4)P pools than previously possible. P4Mx2 increases signal intensity while maintaining spatial specificity, making it particularly useful for live‐cell imaging applications [19, 39, 42].

Overall, PI(4)P serves as a paradigmatic example of how phosphoinositide probes must balance affinity, specificity, and biological compatibility. New generations of probes continue to shed light on the multifaceted roles of PI(4)P across cellular membranes.

### Fluorescent probes for phosphatidylinositol 5‐phosphate [PI(5)P]

PI(5)P is present in a relatively low abundance compared with other phosphoinositides, but is increasingly recognized as being involved in nuclear signaling, endosomal trafficking, and cellular stress responses [43, 44, 45]. Its scarcity has made detection and live‐cell imaging technically challenging.

The most commonly used probe for PI(5)P is the plant homeodomain (PHD) finger from the nuclear protein inhibitor of growth protein 2 (ING2), commonly referred to as ING2‐PHD, which has been reported to bind PI(5)P with some specificity [46, 47]. Using this domain, PI(5)P has been visualized not only in the nucleus but also at the plasma membrane, providing evidence for its functional roles beyond the nucleus [46]. However, ING2‐PHD also interacts with other phosphoinositides and chromatin‐associated factors, which may complicate interpretation, particularly in live‐cell imaging [46].

Alternative approaches have investigated the PH domain of T‐lymphoma invasion and metastasis 1 (Tiam1), a Rho guanine nucleotide exchange factor that has shown selective binding to PI(5)P under certain conditions [48]. Tiam1‐PH has been observed at early endosomes in association with Rac1, suggesting a potential role for PI(5)P in actin cytoskeleton regulation during endocytosis. However, the specificity and utility of Tiam1‐PH as a PI(5)P probe remain under debate, and further validation is needed before it can be universally applied in imaging studies.

Despite these ongoing efforts, PI(5)P remains among the most challenging phosphoinositides to visualize in living cells. Continued probe development will be essential to improve specificity, signal‐to‐noise ratio, and biological compatibility for reliable imaging and mechanistic studies.

### Fluorescent probes for phosphatidylinositol 3,4‐bisphosphate [PI(3,4)P_2_]

PI(3,4)P_2_ is a transient signaling lipid primarily generated by class I PI3‐kinases through the dephosphorylation of PIP_3_ by Src homology 2 (SH2) domain‐containing inositol polyphosphate 5‐phosphatase (SHIP) phosphatases [49, 50]. It plays essential roles in lamellipodia formation, clathrin‐mediated endocytosis, and regulation of the Akt signaling pathway.

The most widely used probe for PI(3,4)P_2_ is the PH domain of tandem PH‐domain‐containing protein 1 (TAPP1; also known as pleckstrin homology domain‐containing family A member 1), which binds selectively to PI(3,4)P_2_ [51]. TAPP1‐PH–GFP constructs allow visualization of this lipid species at the plasma membrane and endosomes [52, 53]. However, compared with other PI‐binding domains, the relatively low affinity of the TAPP1 PH domain for PI(3,4)P_2_ [10], along with its potential for protein–protein interactions, may complicate data interpretation.

Another frequently used probe was the PH domain of Akt, which binds both PI(3,4)P_2_and PIP_3_, thereby limiting its specificity [54, 55]. As a result, the Akt‐PH domain is generally avoided in studies specifically focused on PI(3,4)P_2_.

To overcome these limitations, recent innovations have introduced high‐avidity biosensors employing tandem TAPP1 PH domains, significantly enhancing localization specificity and sensitivity. Notably, a study by Goulden *et al*. [20] using a triple tandem PH domain construct (3xTAPP1‐PH) demonstrated that PI(3,4)P_2_at the plasma membrane is predominantly produced downstream of class I PI3‐kinase signaling.

**Fig. 2:**
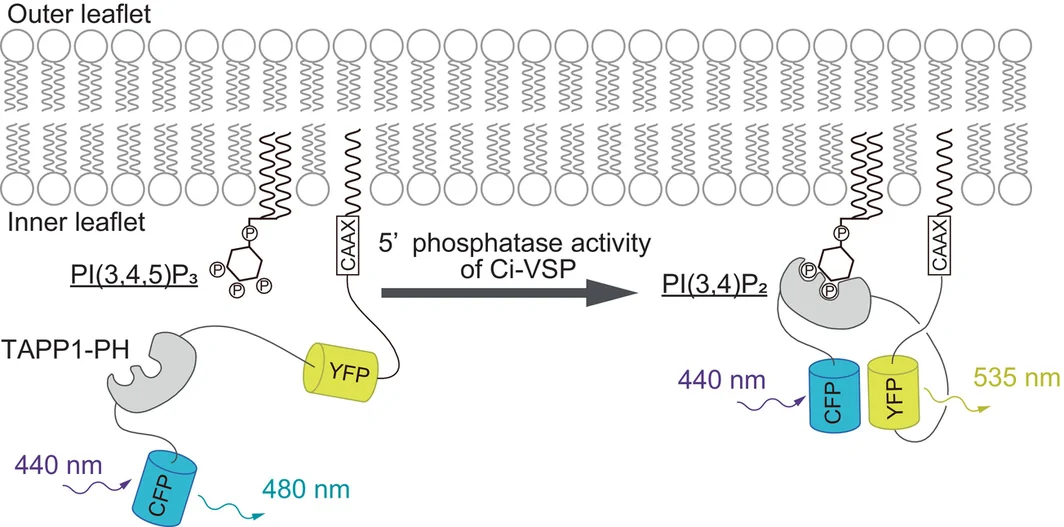
Cartoon depicting a FRET probe for PI(3,4)P_2_. f‐TAPP is a Förster resonance energy transfer (FRET)‐based biosensor for phosphatidylinositol 3,4‐bisphosphate [PI(3,4)P_2_]. The probe is composed of cyan fluorescent protein (CFP), the pleckstrin homology of tandem PH‐domain‐containing protein 1 (TAPP1‐PH), yellow fluorescent protein (YFP), and a CAAX prenylation motif that anchors the construct to the plasma membrane. Under basal conditions, f‐TAPP emits fluorescence at 480 nm upon excitation at 440 nm, even in the presence of phosphatidylinositol 3,4,5‐trisphosphate (PIP_3_, left). Upon activation of *Ciona intestinalis* voltage sensor‐containing phosphatase (Ci‐VSP), whose 5‐phosphatase activity converts PIP_3_ to PI(3,4)P_2_, f‐TAPP can bind the newly generated PI(3,4)P_2_. This binding induces a conformational change that brings CFP and YFP into proximity, resulting in FRET. In this state, excitation at 440 nm leads to emission at 535 nm (right).

Several groups have developed Förster resonance energy transfer (FRET)‐based biosensors utilizing the TAPP1 PH domain to monitor PI(3,4)P_2_ dynamics in live cells. For example, Grimm and Isacoff reported a TAPP1‐PH‐based FRET sensor (f‐TAPP) for visualizing spatiotemporal changes of PI(3,4)P_2_in living cells (Fig. 2) [56]. Similarly, Yoshizaki and colleagues [57] developed another PI(3,4)P_2_ FRET sensor, Pippi‐PI(3,4)P_2_. These tools can be applied in conventional fluorescence microscopy as well as be extended to FRET readouts. More recently, a probe called InPTApp, also based on the TAPP1‐PH domain, was developed to monitor PI(3,4)P_2_at both the plasma membrane and lysosomes [58].

Despite these advances, imaging PI(3,4)P_2_ remains technically challenging due to its low abundance and rapid metabolic turnover. Continued optimization of probes aims to improve signal‐to‐noise ratios and enhance discrimination from closely related phosphoinositide species.

### Fluorescent probes for phosphatidylinositol 4,5‐bisphosphate [PI(4,5)P_2_]

PI(4,5)P_2_is one of the most abundant phosphoinositides, predominantly localized at the plasma membrane, where it regulates cytoskeletal dynamics, membrane trafficking, and signal transduction [59, 60]. The most commonly used probe is the PH domain of phospholipase C delta‐1 (PLCδ1‐PH), which binds PI(4,5)P_2_ with high specificity and has been instrumental in visualizing its dynamics at the plasma membrane [21, 22, 23].

Using PLCδ1‐PH, researchers have revealed the depletion of PI(4,5)P_2_ upon cellular stimulation followed by recovery mediated by PI kinase activity. However, PLCδ1‐PH has limitations in detecting PI(4,5)P_2_ pools in intracellular organelles. Although PI(4,5)P_2_ is reported on the Golgi and endosomes, PLCδ1‐PH often fails to detect these pools, likely due to differences in membrane environment or competition with endogenous proteins.

Alternative probes, such as the GFP‐fused ENTH domain of epsin‐1 (ENTH‐GFP), have been developed to address these issues [24]. The ENTH domain binds PI(4,5)P_2_with high affinity and distinct membrane interaction properties. Therefore, the alternative probe complements PLCδ1‐PH and offers the potential for detecting PI(4,5)P_2_not only at the plasma membrane but also on intracellular organelles.

In addition to protein‐domain‐based sensors, more sophisticated quantitative probes have been developed. Cho *et al*. reported ratiometric fluorescent probes for PI(4,5)P_2_and PIP_3_ using solvatochromic fluorophores, such as 2‐dimethylamino‐6‐acyl‐naphthalene (DAN), (E)‐N′‐((7‐(diethylamino)‐9,9‐dimethyl‐9H‐fluoren‐2‐yl)methylene)acrylohydrazide (WCB), and 3‐((acryloyloxy)imino)‐2‐((7‐(diethylamino)‐9,9‐dimethyl‐9H‐fluoren‐2yl)methylene)‐2,3‐dihydro‐1H‐inden‐1‐one (WCR), enabling ratio‐based readouts of lipid dynamics [61, 62]. In other approaches, Jin Zhang et al. developed FRET‐based and dimerization‐dependent fluorescent‐protein (ddFP)‐based biosensors for PI(4,5)P_2_ and PIP_3_, providing an improved dynamic range and sensitivity for live‐cell imaging [63]. More recently, Datta *et al*. reported cell‐permeable ratiometric peptide‐based probes for PI(4,5)P_2_, enabling visualization of lipid dynamics in living neurons and multicellular organisms [64]. These sensors are advantageous in that they can be delivered exogenously into intact cells, although their selectivity for PI(4,5)P_2_ over PIP_3_ remains to be fully established. The development of more selective variants of this class is therefore anticipated. These strategies complement domain‐based probes by enabling quantitative and real‐time monitoring of lipid levels in living cells.

### Fluorescent probes for phosphatidylinositol 3,5‐bisphosphate [PI(3,5)P_2_]

PI(3,5)P_2_ is a low‐abundance, rapidly turned‐over phosphoinositide predominantly localized to endolysosomal membranes [65]. It is synthesized by phosphoinositide kinase, FYVE‐type zinc finger containing (PIKfyve) from PI(3)P in mammalian cells and Fab1 in yeast [66, 67], and plays a vital role in endosome maturation, lysosomal trafficking, and osmotic stress responses.

One of the earliest and most widely used probes was the cytosolic N‐terminal polybasic region of the transient receptor potential cation channel, mucolipin subfamily (TRPML1; also known as mucolipin‐1), referred to as ML1N [68]. Tandem repeats of ML1N (e.g., ML1N2) tagged with fluorescent proteins localize primarily to endosomal compartments in mammalian and plant cells [68, 69]. However, GFP–ML1N2 localization is not significantly altered in PI(3,5)P_2_‐depleted cells [70]. This finding indicates that the probe does not reflect the intracellular localization of PI(3,5)P_2_ and, therefore, caution is required when interpreting its distribution as a readout of PI(3,5)P_2_ levels.

Recently, the sorting nexin A (SnxA) PX domain from *Dictyostelium discoideum* has been introduced as a new live‐cell probe for PI(3,5)P_2_ [25], offering several advantages. The SnxA PX domain displays high affinity for PI(3,5)P_2_ [with a dissociation constant (*K*d) of ~187 nm], negligible cross‐reactivity with other PIs, and rapid, reversible membrane association upon PIKfyve inhibition. It enables high‐contrast imaging and has revealed previously unknown aspects of PI(3,5)P_2_ dynamics, including pathway‐specific accumulation and asymmetric distribution during cell division in complex with autophagy‐related protein 18 (Atg18) [26], suggesting functional compartmentalization of PI(3,5)P_2_within endolysosomal membranes.

### Fluorescent probes for phosphatidylinositol 3,4,5‐trisphosphate [PI(3,4,5)P_3_]

PI(3,4,5)P_3_ (commonly referred to as PIP_3_) is a critical second messenger involved in cellular growth, metabolism, and survival [71]. It is produced by class I PI3Ks in response to growth factor receptor activation and serves as a key regulator of downstream pathways, including Akt, serine/threonine‐protein kinase mTOR, and Rac.

To monitor PIP_3_ dynamics, several fluorescent biosensors have been developed. Among them, the PH domain of the general receptor for 3‐phosphoinositides 1 (Grp1) is frequently used due to its high selectivity for PIP_3_ over PI(3,4)P_2_ [27]. Grp1 was originally characterized as a guanine nucleotide exchange factor (GEF) for ADP‐ribosylation factor 6 (ARF6) [72], and its PH domain has since been shown to bind PIP_3_ with high affinity. GFP–Grp1‐PH constructs reliably translocate to the plasma membrane upon PI3K activation and are used to trace spatiotemporal changes in PIP_3_ levels with high resolution. Grp1‐PH is further exploited as a FRET‐based PIP_3_ biosensor, named InPGrp, which can detect PIP_3_ at the plasma membrane and lysosomes [58]. Another frequently used probe is the PH domain of Bruton's tyrosine kinase (Btk). Btk‐PH–GFP also shows strong specificity toward PIP_3_ and reduced binding to PI(3,4)P_2_. Similar to GFP–Grp1‐PH, Btk‐PH–GFP has been instrumental in visualizing the accumulation of PIP_3_ at the plasma membrane [22, 28].

In contrast, the PH domain of Akt, although historically one of the first and most widely used tools for PIP_3_ detection, binds both PIP_3_ and PI(3,4)P_2_ with moderate affinity [54, 55]. This dual specificity significantly limits its usefulness in experiments that require precise discrimination of PIP_3_, particularly in cells where both lipid species are present simultaneously. As a result, the Akt‐PH domain is now generally avoided as a standalone probe for PIP_3_ and has been supplanted by more selective alternatives, such as Grp1‐PH and Btk‐PH.

Recently, cell‐permeable biosensors for PIP_3_, named MFR‐K17H and DAN‐NG‐H12G, have been developed to allow ratiometric quantification [73]. They are polarity‐sensitive fluorene dyes conjugated with partial sequences of mutated gelsolin and neurogranin, respectively. The addition of MFR‐K17H in the culture medium enables visualization of PIP_3_ accumulation at the plasma membrane in response to epidermal growth factor (EGF) stimulation. However, these probes are still in the pilot stages of application, with few studies demonstrating their use. Further studies using these probes will confirm their reliability in live‐cell imaging.

In summary, PIP_3_ is a master regulator of growth and survival signaling pathways. Given its vital role in proliferative signals, its levels must be tightly regulated. This highlights the importance of employing highly specific and sensitive biosensors to accurately visualize and quantify PIP_3_ in living cells. Grp1‐PH and Btk‐PH domains currently offer the best balance between specificity and performance, although emerging technologies, such as FRET biosensors and optogenetic control, will refine our ability to dissect PIP_3_ signaling with high fidelity in the future.

## Quantitative mass spectrometry of phosphoinositides

### Technical challenges and background in the quantification of phosphoinositides

As described above, imaging techniques using fluorescent‐protein‐based probes are extremely useful for analyzing the intracellular localization and dynamics of phosphoinositides. However, these methods lack quantitative capacity and are limited in their ability to accurately evaluate changes in lipid molecular species concentrations or metabolic abnormalities. Since the 1980s, radioactive isotope labeling of cultured cells combined with anion‐exchange chromatography has been widely used for phosphoinositide detection and quantification, but this method requires a simple deacylation step using methylamine, which results in the loss of acyl chain information [74]. To date, no molecular probes capable of distinguishing the acyl chain structures of phosphoinositides have been developed. Furthermore, although radioactive isotope labeling is applicable to cultured cells, it is difficult to apply to clinical specimens or animal tissues. *In vivo* imaging using transgenic mice expressing fluorescent probes has been attempted by our group [75, 76], but such methods have not yet become broadly adopted. Thus, from multiple perspectives—including the need for highly quantitative measurement of phosphoinositides, the analysis of their dynamics in animal tissues and body fluids, and the characterization of individual molecular species—MS‐based analytical approaches offer a promising solution.

### Advances in lipidomics and the analytical challenges of phosphoinositides

Lipidomics has undergone remarkable development over the past two decades as a technology for comprehensively and quantitatively analyzing diverse lipid molecules in biological systems. Using MS, numerous platforms have been established to sensitively and simultaneously analyze a broad range of structurally and functionally diverse lipid classes, including glycerophospholipids, sphingolipids, steroids, fatty acids and their derivatives, and neutral lipids [77, 78, 79].

Among glycerophospholipids, phosphatidylcholine (PC), phosphatidylethanolamine (PE), phosphatidylserine (PS), and PI can be efficiently extracted using standard organic solvents and separated by reversed‐phase chromatography according to their fatty acid composition. These lipids also exhibit high ionization efficiency in negative ion mode, making them ideal targets in lipidomics research due to their robust quantitative performance.

In contrast, phosphoinositides are rarely detected by conventional nontargeted lipidomic platforms. Several factors contribute to this difficulty. First, phosphoinositides are strongly acidic lipids bearing multiple phosphate groups, resulting in poor extraction efficiency under standard organic‐solvent‐based protocols. Second, their abundance in total lipid extracts from cells, tissues, or blood is extremely low, rendering them highly susceptible to ion suppression during MS. Third, phosphoinositides are prone to sample loss during preparation, which compromises analytical accuracy and reproducibility.

Due to these technical barriers, phosphoinositides have long remained a ‘blind spot’ in lipidomics. However, in recent years, dedicated targeted lipidomics approaches have been developed to overcome these challenges. By optimizing extraction, separation, and detection strategies tailored to the unique chemical properties and metabolic characteristics of phosphoinositides, it has become possible to distinguish structural isomers and quantify even trace‐level phosphoinositides with high precision. These developments are outlined in the following sections.

### Improved sensitivity through refinement of extraction and chromatography methods

Early studies employed direct infusion MS for the analysis of phosphoinositides; however, issues, such as ion suppression and complex mixed spectra became apparent [80, 81]. Subsequent advances have been achieved by optimizing lipid extraction protocols and chromatographic separation techniques during sample preparation. Standard protocols based on the Bligh and Dyer or Folch methods yield lower recovery rates for highly polar and strongly acidic lipids, such as phosphoinositides, compared with other lipid classes [82, 83]. To address this limitation, extraction under acidic conditions—intended to protonate phosphate groups and improve solubility in organic solvents—has been explored. Nevertheless, for highly phosphorylated phosphoinositides, extraction using hydrochloric acid (HCl) has been reported to result in low recovery and poor reproducibility due to hydrolysis in the aqueous phase remaining after biphasic separation [84].

Pettitt *et al*. also developed an improved extraction protocol that minimized acid hydrolysis and employed normal‐phase liquid chromatography using silica columns to separate positional isomers of PI(4,5)P_2_ [84]. They further reported that quantitative estimation of PI(3)P and PI(4)P—excluding PI(5)P—could be achieved by assessing the relative intensities of diacylglycerol‐derived fragments generated during MS^3^ fragmentation [84].

Milne *et al*. [81] later proposed an extraction method in which neutral lipids were first removed using neutral solvents, followed by selective extraction of phosphoinositides in acidic methanol. This approach enabled efficient detection and quantification of PIP_3_ in biological samples. Using this method, they identified 28 molecular species of phosphatidylinositol mono‐ and bisphosphates and eight PIP_3_ species with distinct acyl chain compositions from primary mouse macrophages and demonstrated stimulus‐dependent production of specific PIP_3_ species.

Furthermore, Ogiso *et al*. [85] proposed a method combining diethylaminoethyl (DEAE) cellulose‐based enrichment of acidic phospholipids with reversed‐phase liquid chromatography. These studies collectively demonstrate that improvements in extraction and separation procedures have laid a critical foundation for the high‐sensitivity, high‐selectivity quantification of phosphoinositides by MS.

### Improved recovery and stability through derivatization: the TMS‐diazomethane method

Clark *et al*. [86] developed a methylation‐based derivatization method using trimethylsilyl (TMS)‐diazomethane to achieve highly sensitive and accurate quantification of phosphoinositides. In this approach, phosphate groups on phosphoinositides are quantitatively methylated, with three, five, and seven methyl groups introduced under optimized conditions for PI monophosphate, bisphosphate, and trisphosphate species, respectively. This derivatization markedly reduces the polarity of these lipids, thereby enhancing their retention in reversed‐phase chromatography and dramatically increasing ionization efficiency in positive ion mode. Compared with other derivatization techniques, the TMS‐diazomethane method is superior in terms of operational simplicity, reaction speed, chemical stability, and specificity—an advantage confirmed by multiple studies, including those from our group.

Leveraging this derivatization technique, Clark *et al*. [86] performed detailed fatty acid composition analysis of phosphoinositides extracted from biological samples, such as platelets. They quantitatively tracked the increase in PIP_3_ species following cellular stimulation and reported stimulus‐responsive shifts in phosphoinositide molecular species. Using this same method, the group also demonstrated that, in *Dictyostelium*, plasmanylinositols—phosphoinositides containing an ether‐linked C16:0 chain at the stereospecific numbering (*sn*)‐1 position—are abundant and act as functional lipids that regulate chemotaxis [87].

### Analysis of acyl chain positional information and chemical modifications

Variations in the fatty acid composition at the sn‐1 and sn‐2 positions of the glycerol backbone can give rise to structural isomers at the molecular species level. In many studies, the total number of carbon atoms and double bonds in the acyl chains is reported as a sum of the fatty acids at both positions. However, Kim *et al*. [88] introduced a method for determining the positional assignment of these acyl chains. In their approach, methylated derivatives of phosphoinositides are separated by reversed‐phase liquid chromatography and analyzed by collisionally activated dissociation (CAD). Monoacylglycerol (MAG) fragments generated during CAD serve as diagnostic ions for identifying the specific composition of fatty acids at the sn‐1 and sn‐2 positions. Specifically, the mass‐to‐charge ratio of MAG fragments, formed after neutral loss of a fatty acid from the [M–H]^−^ ion, is used to assign the positional identity of each acyl chain.

It is also known that the hydrocarbon chains of acyl groups in phospholipids are susceptible to nitration and nitro‐oxidation. Bonciarelli et al. recently reported a method for analyzing structural modifications of PI induced by *in vitro* treatment with nitronium tetrafluoroborate (NO_2_BF₄) [89]. Using C18 reversed‐phase liquid chromatography coupled with electrospray ionization (ESI)‐MS/MS, they characterized nitro‐ and nitro‐oxidized PI species. The MS/MS spectra revealed diagnostic fragment ions, including a characteristic neutral loss of HNO_2_ (47 atomic mass units), carboxylate anions of nitrated fatty acids, and composite fragmentations involving the inositol head group. These features allowed for the identification of positional isomers of chemically modified phosphoinositides.

### Diverse strategies for distinguishing phosphorylation positional isomers

Phosphorylation positional isomers of PI monophosphate and bisphosphate species possess highly similar chemical structures and identical molecular masses, making it difficult to achieve sufficient retention differences using conventional reversed‐phase liquid chromatography. To address this limitation, efforts have focused on optimizing chromatographic conditions to enable physical separation, along with innovations in mass spectral acquisition and interpretation.

One approach involves deacylation of lipid extracts by methylamine to produce glycerophosphoinositol phosphates, which are subsequently detected using a combination of anion‐exchange chromatography and selected reaction monitoring (SRM). This method enables sensitive quantification of the three positional isomers of phosphatidylinositol bisphosphate [90]. Another strategy, developed by Malek *et al*., utilizes ozonolysis to cleave the carbon–carbon double bonds in the acyl chains of PI(3,4)P_2_ and PI(4,5)P_2_, shortening the chains and enhancing retention time differences on a C18 amide column in reversed‐phase chromatography [91].

Additionally, a sophisticated method has been reported in which positional isomers of PI(3)P and PI(4,5)P_2_are differentiated based on distinct methylation patterns arising from trimethylsilyl diazomethane treatment of phosphate groups [92]. In this workflow, shotgun lipidomics is used to acquire fragment spectra, and an algorithm is applied to quantify relative abundances of positional isomers in mixed samples. This allows PI(3)P to be distinguished from PI(4)P and PI(5)P, as well as enabling specific quantification of each PI(4,5)P_2_isomer.

**Fig. 3:**
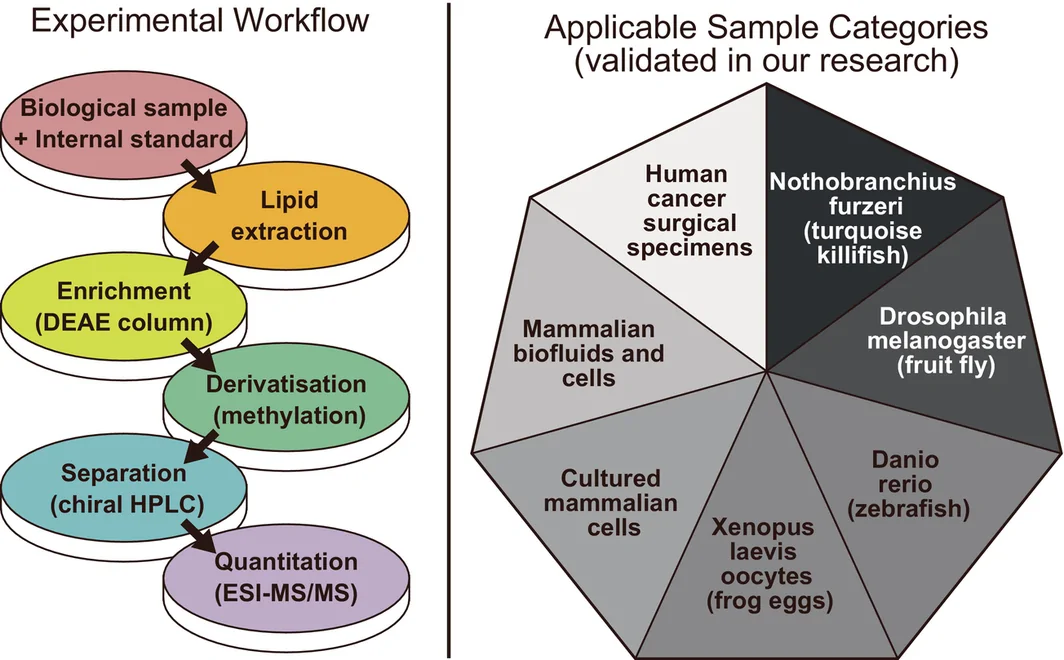
General workflow and applicability of PRMC‐MS (phosphoinositide regioisomer measurement by chiral column chromatography and mass spectrometry). This targeted lipidomics workflow enables sensitive and comprehensive quantification of phosphoinositide variants, including regioisomers, by combining acidified lipid extraction, enrichment by diethylaminoethyl sepharose (DEAE) column, trimethylsilyl diazomethane derivatization, chiral column‐based high performance liquid chromatography (HPLC) separation, and multiple reaction monitoring (MRM) on a triple quadrupole electrospray ionization tandem mass spectrometer (ESI‐MS/MS) (left). Acyl variants are defined by the total carbon number and degree of unsaturation of the fatty acyl chains at the sn‐1 and sn‐2 positions. The method has been validated across diverse biological and clinical specimens, demonstrating broad applicability (right).

Several groups have reported comprehensive methods for the simultaneous analysis of seven phosphoinositide phosphorylation isomers. Shimanaka *et al*. [93] developed a technique combining supercritical fluid chromatography (SFC) using a β‐cyclodextrin column with multiple reaction monitoring (MRM)‐based MS/MS to identify and quantify phosphoinositide molecular species while retaining acyl chain information. Two independent groups have also reported phosphoinositide profiling methods using chiral column chromatography. Li and Lämmerhofer established a comprehensive phosphoinositide workflow by integrating chromatography based on a chiral polysaccharide stationary phase with sequential windowed acquisition of all theoretical fragment ion mass spectra (SWATH‐MS), a data‐independent acquisition (DIA) method that records all detectable components with high reproducibility [94]. Our group developed a method called phosphoinositide regioisomer measurement by chiral column chromatography and mass spectrometry (PRMC‐MS), which combines phosphoinositide enrichment using DEAE cellulose, derivatization with TMS‐diazomethane, chiral chromatographic separation, and MRM‐based detection on a triple quadrupole mass spectrometer (Fig. 3) [95]. Using this method, we routinely perform comprehensive analyses of approximately 200 distinct acyl variants—defined by the total carbon number and degree of unsaturation of the fatty acyl chains at the sn‐1 and sn‐2 positions—across the eight phosphoinositide classes, including phosphatidylinositol and its seven phosphorylated forms. PRMC‐MS is broadly applicable for quantifying phosphoinositide molecular species in a wide variety of biological specimens, including model organisms, such as *Saccharomyces cerevisiae*, *Drosophila melanogaster*, and zebrafish, as well as cultured cells, mouse and human tissues, spermatozoa, plasma, exosomes, purified organelles, and immunoprecipitated protein–phosphoinositide complexes [95, 96, 97, 98, 99, 100, 101].

## Summary and future perspectives

Given the extensive research on phosphoinositides across diverse biological fields, we acknowledge that this review may not include all relevant studies. We apologize for any unintentional omissions.

A comprehensive understanding of phosphoinositide biology requires the integration of both spatiotemporal and quantitative analytical strategies. Fluorescent probes have enabled dynamic visualization of phosphoinositide signaling in live cells, revealing localized changes in lipid distribution in response to various stimuli. However, the design of such probes remains a balance between specificity, sensitivity, and minimal perturbation to endogenous systems. Limitations remain in the development of probes for certain phosphoinositide species, such as PI(5)P and PI(3,5)P_2_, and the influence of fatty acyl chain diversity on probe recognition is still largely unexplored. FRET‐based sensors provide a unique means to monitor low‐abundance lipids in real time, yet current designs are constrained by low dynamic range, limited signal‐to‐noise ratio, and the bulky nature of the sensor, which can compromise accurate tracking of rapid lipid dynamics. Future efforts will need to address these challenges to realize the full potential of FRET‐based approaches.

Complementing these imaging‐based approaches, recent breakthroughs in MS have enabled extremely sensitive and isomer‐resolved quantification of phosphoinositides, overcoming long‐standing analytical challenges related to their low abundance, structural diversity, and ion suppression. The development of optimized extraction protocols, derivatization techniques, and advanced chromatographic platforms—such as chiral LC, SFC, and ion chromatography—has facilitated comprehensive profiling of over 200 distinct molecular species, including positional isomers and acyl variants. These advances have uncovered new biological insights into the compartment‐specific metabolism and extracellular dynamics of phosphoinositides across diverse systems.

Looking ahead, the convergence of spatial and quantitative phosphoinositide analysis may be realized through imaging mass spectrometry (IMS), which enables *in situ* detection of lipid species directly within tissue sections. Although IMS does not allow live‐cell imaging, it uniquely integrates spatial resolution with molecular specificity and quantitative capacity. Currently, the spatial resolution of IMS typically ranges from several to tens of micrometers, which is sufficient to visualize tissue‐ and cell‐type‐specific lipid distributions. With ongoing advancements in ionization efficiency and matrix optimization, further improvements in spatial resolution may enable subcellular or even organelle‐level phosphoinositide mapping. As such, IMS offers a unifying technological framework that bridges the goals of fluorescent‐probe‐based imaging and MS‐based quantification—providing an essential tool for decoding the complex, dynamic landscape of phosphoinositide signaling in health and disease.

## Author contributions

HK, SM, JS, and TS jointly contributed to the writing of the manuscript, including drafting, editing, and revising the text. All authors reviewed and approved the final version of the manuscript.

## Conflict of interest

The authors declare no conflicts of interest.
